# Bdellatomy. Taking Advantage of a Leech

**Published:** 1868-06

**Authors:** 


Bdellatomy. Taking Advantage of a Leech.—A curious practice lately introduced in Germany is the cutting of the leech so that the blood will flow out of his body as fast as he sucks it from the patient. An ounce, or even two ounces, may be drawn in this way by a single leech. The spring lancet is preferred, though a thumb lancet will answer. The incision is made in the side, the left side being preferable, and at the time when the leech has nearly filled himself, and just before he is ready to stop sucking. The wound is kept free from coagulated blood by a warm sponge, or even by injecting warm water into the wound. If from rough handling the leech falls off, it takes hold again without difficulty. The process has been named Bdellatomy (*bdella*, a leech). At first sight it looks like taking an unfair advantage of the animal, if not treating him cruelly. But it is probably just the reverse, as it affords him an opportunity to feast longer on his rich beverage without giving any noticeable pain. If carefully kept in clean water the same leech may be repeatedly applied, and incised at intervals of days or weeks.

